# Nondestructive Localization of Subvisual Defects in Laser-Induced Graphene via Machine-Learning-Assisted Electrical Resistance Tomography

**DOI:** 10.1021/acsomega.6c01789

**Published:** 2026-06-04

**Authors:** Keiya Minakawa, Kotaro Takanashi, Yuki Kimura, Annop Klamchuen, Winadda Wongwiriyapan, Takashi Ikuno

**Affiliations:** † Department of Applied Electronics, Graduate School of Advanced Engineering, 26413 Tokyo University of Science, Katsushika, Tokyo 125-8585, Japan; ‡ National Nanotechnology Center, NSTDA, 111 Thailand Science Park, Klong Luang, Pathum Thani 12120, Thailand; § Department of Nanoscience and Nanotechnology, School of Integrated Innovative Technology, King Mongkut’s Institute of Technology Ladkrabang, College of Materials Innovation and Technology, Ladkrabang, Bangkok 10520, Thailand; ∥ Research Institute for Science and Technology, 26413 Tokyo University of Science, Noda, Chiba 278-8510, Japan

## Abstract

Although conventional imaging techniques excel at capturing structural changes, they frequently overlook functional degradations that lack morphological signatures. Here, we demonstrate machine-learning-assisted electrical resistance tomography (ML-ERT) as a robust modality for the rapid, nondestructive localization of “subvisual” defects in porous laser-induced graphene (LIG). By employing masked O_2_ plasma irradiation, we introduced localized defects that exhibit a dramatic resistance surge up to 4 orders of magnitude while remaining indistinguishable under visual and electron microscopy. Our ML-ERT framework, powered by a one-dimensional convolutional neural network inverse solver, successfully pinpointed these hidden failures once the resistance contrast reached a threshold of *R*/*R*_0_ ≥ 6.71. Furthermore, 3D finite element analysis revealed that the tomographic contrast is driven by an effective conductive volume loss exceeding 30%, identifying the degradation of internal conductive pathways as the primary mechanism. These results establish ML-ERT as a high-sensitivity diagnostic tool capable of visualizing electrically critical but optically invisible failures, providing a definitive solution for the quality control of large-area carbon electronics.

## Graphical abstract

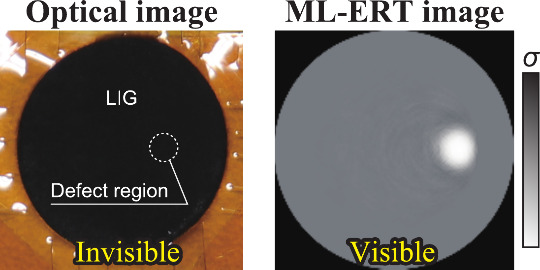

## 1 Introduction

Laser-induced graphene (LIG), produced via direct laser writing on polyimide (PI), has emerged as a versatile carbon material that combines scalable fabrication with a three-dimensional porous microstructure and high electrical conductivity.^1–3^ Owing to these features, LIG has been extensively explored as an active layer in flexible sensors, electrochemical devices, and energy systems.^4–9^ In such applications, device performance and reliability are primarily governed by the continuity of the conductive network.^10,11^ Local defects, process-induced inhomogeneities, or damage accumulated during operation can locally degrade conductivity and trigger significant performance loss.^11–13^ Consequently, identifying the spatial distribution of electrical degradation and assessing its severity are essential for process optimization, quality control, and failure analysis.

Conventional characterization methods provide indispensable information but are not always aligned with the electrical functionality that determines device-scale performance. For instance, while Raman mapping and scanning electron microscopy (SEM) can visualize structural disorder and morphology with high spatial resolution, they require time-consuming spatial scanning that becomes a bottleneck for large-area inspection.^14–16^ Furthermore, many spectroscopic techniques are intrinsically surface-sensitive.^17^ X-ray photoelectron spectroscopy (XPS), for example, primarily probes near-surface chemical states and does not directly quantify the disruption of internal current-carrying pathways within a porous conductor.^17–19^ As a result, regions that appear only subtly modified in surface-sensitive measurements may nonetheless suffer from severe functional degradation if the underlying three-dimensional network is compromised.

While standard electrical tests are rapid and directly relevant to operation, two-terminal or four-terminal measurements provide only spatially averaged information.^20^ These methods can quantify the global resistance increase but fail to localize the degraded region, which limits their utility for diagnosing defects in large-area films. Therefore, a characterization approach capable of pinpointing electrically relevant degradation without resorting to point-by-point scanning would fill a critical gap in the development of LIG-based electronics.

Electrical resistance tomography (ERT) offers a route toward the nondestructive localization of internal conductivity variations through boundary measurements.^21–24^ Since ERT does not require embedded wiring or pixelated sensor arrays, it is particularly attractive for large-area films and irregular geometries.^24–29^ Crucially, ERT is sensitive to the current distribution within the conductor and can thus capture the electrical consequences of internal pathway degradation, even when the modified region remains indistinguishable to optical inspection.^30,31^ Although conventional ERT reconstruction is an ill-posed inverse problem that typically requires complex regularization, recent advancements in data-driven inverse solvers have enabled more stable and accelerated reconstructions by learning the direct mapping from boundary voltage patterns to conductivity distributions.^32–37^

In this work, we demonstrate machine-learning-assisted ERT (ML-ERT) for localizing masked oxygen-plasma-induced defects in porous LIG. Masked plasma irradiation allows for the controllable introduction of localized electrical degradation without producing strong optical contrast, providing an ideal test case for the detection of “subvisual” defects. We characterize the plasma-power-dependent alterations in LIG via Raman spectroscopy, SEM, and electrical measurements, and subsequently utilize an eight-electrode ERT system to reconstruct two-dimensional conductivity maps. A one-dimensional convolutional neural network (1D-CNN) is trained on simulated data sets to serve as the inverse solver. Finally, to interpret the physical origin of the tomographic contrast, we perform a 3D finite element analysis that treats the plasma modification as an effective loss of current-carrying cross-section. The combined results establish a practical framework that complements conventional surface analysis for the rapid, nondestructive localization of electrically relevant defects in LIG.

## 2 Methods

### 2.1 Fabrication of LIG

PI films with the thickness of 100 μm (Changzhou Jinlong Insulation Material Co., Ltd., China) were prepared as substrates.^38^ LIG was synthesized on these PI films using CO_2_ laser (Trotec Speedy 300, TROTEC Laser GmbH, Austria) with a wavelength of 10.6 μm, as illustrated in Figure 1a. The laser irradiation was performed under ambient conditions. To achieve a high degree of graphitization and uniform sheet resistance,^39,40^ the laser parameters were optimized with a power of 4.8 W and a scanning speed of 11.2 cm/s, maintaining a constant focal distance of 25 mm. The LIG was patterned into a circular geometry with a diameter of 14 mm, centered on the PI film, to serve as the active sensing domain for subsequent tomography analysis (Figure 1b). The structural and electrical uniformity of the as-prepared LIG discs were verified to ensure a consistent baseline prior to the defect induction process.

**Figure 1:**
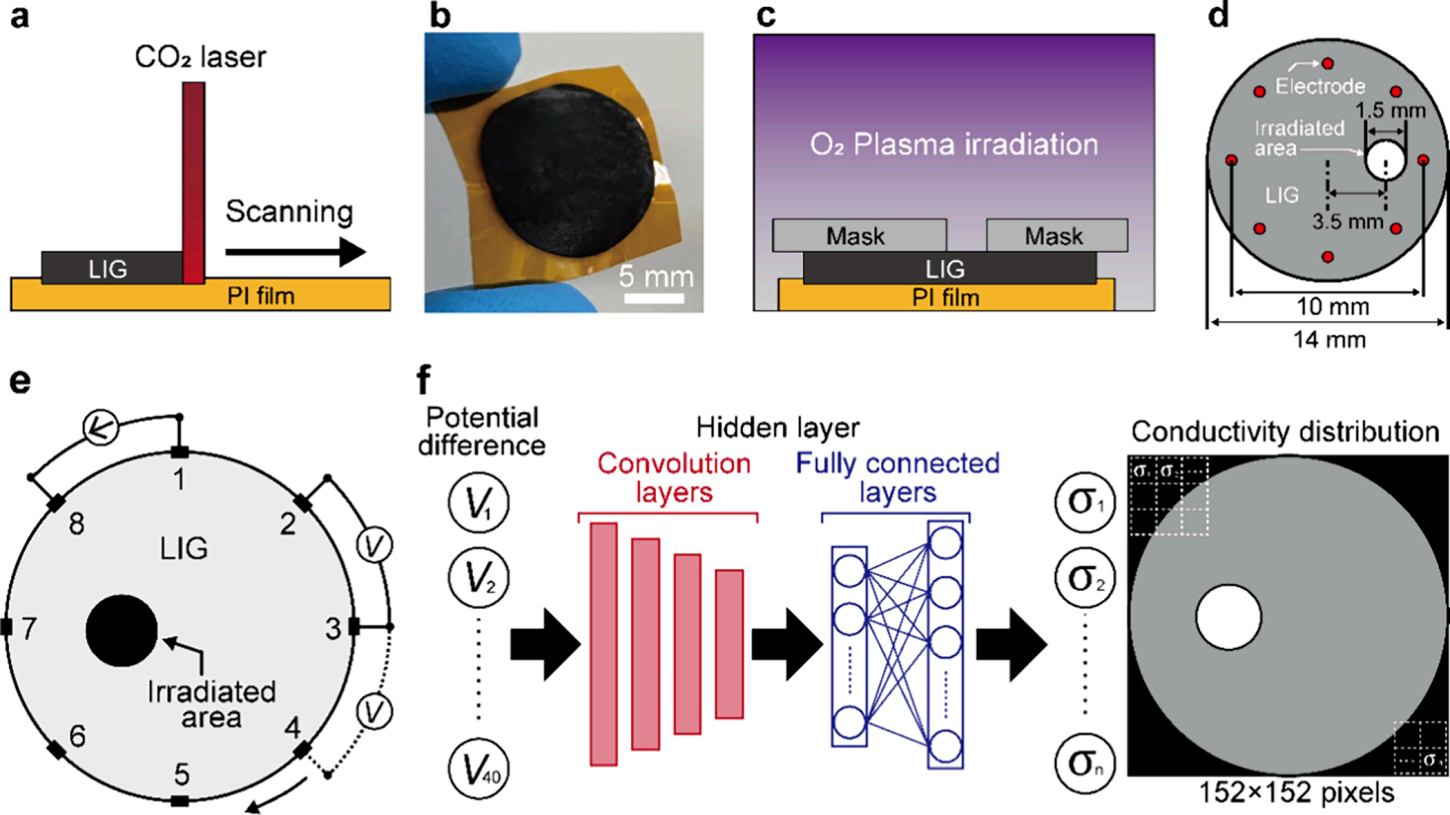
Methodology for Laser-Induced Graphene (LIG) Fabrication, Defect Induction, and Machine Learning-Based Electrical Resistance Tomography (ML-ERT). (a) Schematic illustration of the direct CO_2_ laser-writing process on a PI film to synthesize LIG. (b) Photograph of a fabricated circular LIG sample (diameter: 14 mm) on a PI substrate. (c) Localized oxygen plasma irradiation through a shadow mask with a circular aperture to introduce subvisual defect regions. (d) Geometric configuration of the eight spring-loaded electrodes and the localized irradiation area positioned 3.5 mm off-center. (e) Sequential adjacent-drive measurement scheme used to acquire 40 independent boundary voltage-difference data points. (f) Architecture of the ML reconstruction pipeline, where a one-dimensional convolutional neural network (1D-CNN) maps the input voltage vector to a high-resolution (152 × 152 pixels) two-dimensional conductivity distribution.

### 2.2 O_2_ Plasma Irradiation for Defect Induction

Localized defects were introduced into the LIG films via oxygen plasma irradiation using a capacitively coupled plasma system (PR200, Yamato Scientific Co., Ltd., Japan), as depicted in Figure 1c. High-purity O_2_ gas was employed as the process gas. The plasma exposure duration was maintained at 3 min, while the radio frequency (RF) power was systematically varied between 50 and 150 W to modulate the defect severity (i.e., the degree of local oxidation and conductivity reduction).^41,42^ To evaluate the spatial resolution and localization accuracy of the ERT framework used in this work, an off-center defect configuration was intentionally fabricated (Figure 1d). A shadow mask with a circular aperture (1.5 mm diameter) was positioned 3.5 mm off-center (toward the right hemisphere of the LIG disk) to define the plasma-exposed region. This asymmetric positioning allowed for a rigorous assessment of the model’s capability to reconstruct noncentrosymmetric conductivity distributions.

### 2.3 Electrical Characterization

To quantify the electrical impact of O_2_ plasma-induced defects, current–voltage (*I*–*V*) measurements were conducted using strip-shaped LIG samples (3 × 10 mm^2^). These samples were fabricated using the same laser-writing parameters as the LIG discs described above to ensure consistency in the material properties. O_2_ plasma was irradiated onto a 3 × 3 mm^2^ area at the center of each strip for 3 min at RF powers ranging from 50 to 150 W. For electrical contact, silver paste (DOTITE D-500, Fujikura Kasei Co., Ltd., Japan) was applied to both longitudinal ends.

*I*–*V* characteristics were measured under ambient conditions using a source meter (2612A, Keithley Instruments, Inc., USA) integrated with a probe station (MBP-55-TRI, Apollo Wave Inc., Japan). The bias voltage was swept from −5 to +5 V. All samples exhibited ohmic behavior, and the resistance (*R*) was extracted from the linear slope of the *I*–*V* curves. Three independent samples were characterized for each RF power condition to ensure statistical reliability. The defect-induced resistance change was evaluated using the ratio *R*/*R*_0_, where *R*_0_ and *R* represent the resistances before and after plasma irradiation, respectively.

### 2.4 Structural and Morphological Characterization

Raman spectroscopy was utilized to evaluate the evolution of structural defects and the graphitic quality of the LIG samples following O_2_ plasma irradiation. Spectra were acquired using a micro-Raman spectroscope (inVia Reflex, Renishaw plc, UK) equipped with a 532 nm-excitation laser. The D, G, and 2D peaks were analyzed to quantify the degree of oxidation and lattice disorder induced by the plasma treatment.^43,44^

Surface morphology was observed via field-emission scanning electron microscopy (FE-SEM; GeminiSEM 360, Carl Zeiss Microscopy Deutschland GmbH, Germany) at an accelerating voltage of 5 kV. To investigate the potential structural degradation or etching effects, images were captured at multiple magnifications using an in-lens secondary electron detector. This morphological analysis was crucial for confirming that the plasma-induced resistance changes originated from chemical/electronic modifications rather than macroscopic structural ablation.

### 2.5 ERT Measurement Configuration

To collect boundary potential data for tomographic reconstruction, an ERT measurement system equipped with eight spring-loaded probe electrodes was employed. The electrodes were arranged at equidistant intervals around the circumference of the 14 mm diameter LIG active area, as illustrated in Figure 1e. To ensure stable electrical contact with the porous LIG network, a constant mechanical pressure was applied through the probe array.

The adjacent current-injection protocol (adjacent-drive scheme)^25^ was implemented using a 10 mA sinusoidal AC current at a frequency of 1 kHz. For each pair of adjacent current-injection electrodes, the resulting voltage differences were sequentially recorded between all remaining adjacent electrode pairs. This measurement cycle was repeated for all eight injection pairs, yielding a total of 40 independent voltage-difference measurements per sample (8 × 5 = 40). These 40 features were then utilized as the input vector for the machine learning-based conductivity reconstruction.

### 2.6 ML-Based Reconstruction Method

To reconstruct the two-dimensional conductivity distribution from the boundary potential data, an inverse model based on a 1D-CNN was utilized. The 1D-CNN architecture was chosen to effectively extract spatial features from the sequential ERT voltage measurements.^23,37,45,46^ The network consists of stacked 1D convolutional layers for feature extraction, followed by fully connected layers that map the high-dimensional features to a pixelated conductivity map.

For model training, a comprehensive data set of 14,976 synthetic samples was generated via numerical simulations. Each synthetic case modeled a localized circular defect (conductivity σ = 200 S/m) within a uniform conductive background (σ = 2000 S/m). For each case, the 40-element boundary voltage-difference vector was computed using a finite element method (FEM) framework that replicated the eight-electrode adjacent-drive protocol (Figure 1f). To enhance the model’s robustness against experimental scaling differences, each voltage vector was normalized by its minimum value. The model was trained for 100 epochs with a batch size of 60. The full synthetic data set was used for training, and the training behavior was monitored using MSE and MAE over epochs (Figure S1). No separate validation or test data set was used in the present study. Finally, the experimentally acquired voltage vectors were fed into the trained 1D-CNN to reconstruct 2D conductivity maps, enabling the subvisual defect regions in the LIG samples to be visualized.

### 2.7 ERT Simulation Using Finite Elemental Method

The 3D FEM model was constructed to demonstrate that the contrast observed in ERT could be attributed to a reduction in the current path (effective cross-section) at the defect location. The model represented a 14 mm diameter LIG with the same 8-electrode configuration as the experiment in 3D, with a film thickness of 50 μm. The defect was modeled as a circular region with a diameter of 1.5 mm, offset 3.5 mm to the right from the center, identical to the one introduced in the experiment. Only the defect region had its film thickness locally reduced. The forward problem was solved using 3D FEM, and 40 voltage difference data points were obtained following the same adjacent voltage application and adjacent voltage difference acquisition procedure as in the experiment. The resulting 40-dimensional vector was then fed into the ML model trained on 2D simulated data, and a 2D resistance map was reconstructed to evaluate whether thickness reduction alone (i.e., reduced effective current pathways) can reproduce the defect contrast.

## 3 Results and Discussion

### 3.1 Electrical Characteristics of Plasma-Irradiated LIG

To establish a quantitative foundation for defect detection, we first investigated the modulation of LIG’s electrical properties under varying oxygen plasma intensities using strip-shaped samples. Figure 2a presents representative *I*–*V* curves for pristine LIG and samples irradiated at various plasma powers. All curves exhibited strictly linear, ohmic behavior within the −5 to +5 V range, confirming that the plasma treatment modulates the carrier transport properties of the carbon network without introducing rectifying junctions or nonohmic contact barriers. However, the slope of the *I*–*V* curves decreased significantly with increasing plasma power, reflecting a controllable suppression of conductivity. The extracted resistance values increased from 27.0 Ω for the pristine LIG to 167 Ω at 100 W, eventually reaching 3.72 kΩ at 125 W.

**Figure 2:**
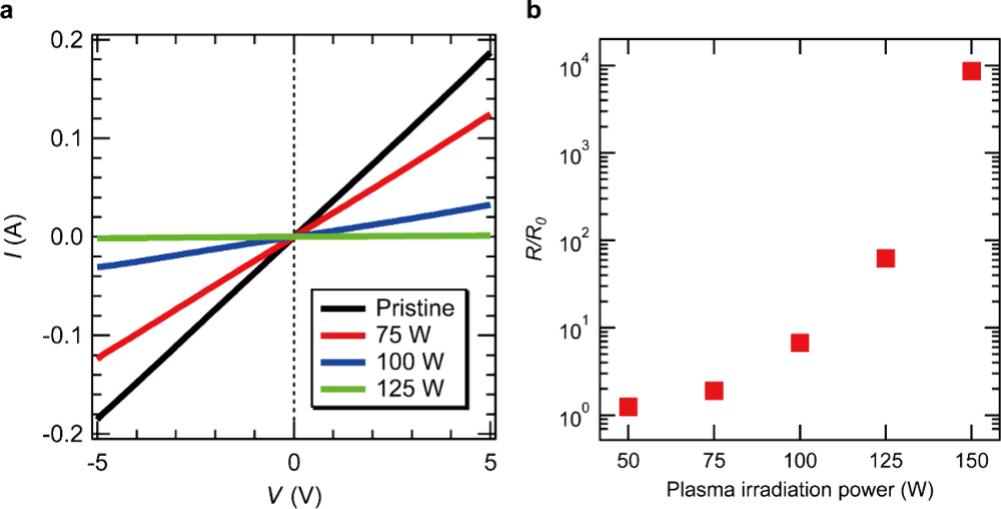
(a) Representative current–voltage (*I*–*V*) curves for pristine LIG and plasma-irradiated LIG (at selected plasma powers), measured over a bias range of −5 to +5 V. (b) Resistance-change ratio (*R*/*R*_0_) as a function of plasma irradiation power.

The evolution of the resistance-change ratio, *R*/*R*_0_, reveals a distinct transition in the damage regime induced by the O_2_ plasma (Figure 2b). At a lower plasma power of 75 W, the resistance exhibited a relatively subtle increase, reaching approximately double its initial value (*R*/*R*_0_ = 1.91). Notably, at 100 W the resistance-change ratio increased to *R*/*R*_0_ = 6.71, marking the onset of the rapid rise observed at higher plasma powers. However, beyond 100 W, the network entered a regime of exponential degradation; the ratio surged to 62.4 at 125 W and culminated in a nearly four-order-of-magnitude increase (*R*/*R*_0_ = approximately 8.67 × 10 ^3^) at 150 W. This highly nonlinear trend suggests that the plasma treatment can precisely tune the LIG from a highly conductive state to a near-insulating phase.

Crucially, this wide dynamic range of conductivity contrast is essential for evaluating the sensitivity of the ML-ERT framework. While the high-contrast regions formed at 125 and 150 W are expected to be easily detectable, the subtle electrical variations at lower powers (≤75 W) pose a much more rigorous challenge for tomographic localization. The ability to distinguish such a broad spectrum of electrical damage, particularly in the absence of obvious structural changes (as discussed in the following section), provides a compelling validation for the proposed ML-ERT approach.

### 3.2 Plasma-Induced Structural Changes

To correlate the observed resistance surge with structural alterations, we analyzed the samples using Raman spectroscopy and SEM Figure 3a displays the Raman spectra, where the prominent D, G, and 2D bands confirm the high-quality graphitic framework of the pristine LIG. Upon plasma exposure, the *I*_G_/*I*_D_ ratio decreased from 1.54 (pristine) to 0.74 at 100 W, signifying a substantial introduction of structural disorder and oxygen-containing functional groups into the graphitic lattice. This chemical and electronic perturbation of the sp^2^ hybridized network is the primary reason for the conductivity suppression observed in Figure 2. At 50 W, the *I*_G_/*I*_D_ ratio showed only a slight increase, which was not considered sufficient to indicate a meaningful improvement in crystallinity, given the inherent structural nonuniformity of porous LIG.

**Figure 3:**
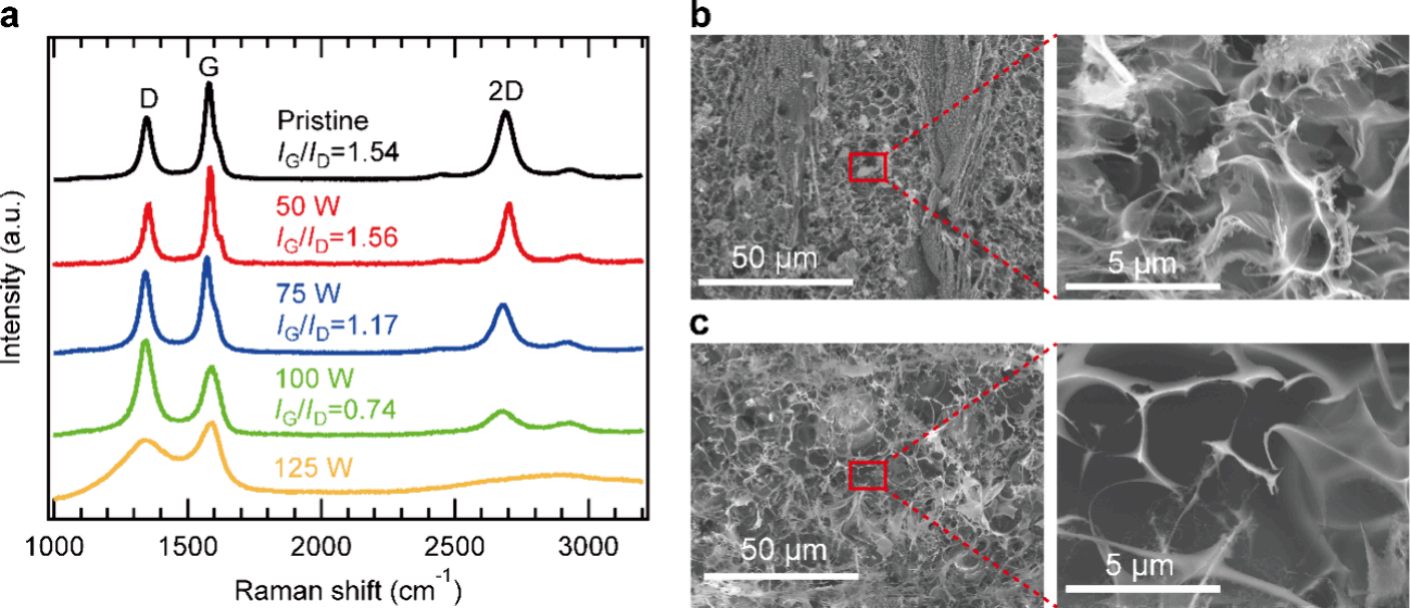
(a) Raman spectra of pristine LIG and plasma-irradiated LIG (peaks for D, G, and 2D bands are labeled), showing the evolution of structural disorder with increasing plasma power. (b) Field-emission SEM images of pristine LIG at low and high magnifications. (c) Field-emission SEM images of the plasma-irradiated region (100 W) at low and high magnifications, where localized etching of the porous LIG framework becomes evident at higher magnification.

The morphological impact of this plasma treatment was examined via SEM. At low magnification, the plasma-exposed region at 100 W is visually indistinguishable from the pristine area, with no macroscopic ablation or boundary lines observed. Higher-magnification images (Figure 3b,c) reveal that the characteristic 3D porous network and flake-like morphology of the LIG are remarkably well-preserved even after 100 W irradiation. While subtle thinning of the carbon flakes can be detected upon rigorous inspection, the overall interconnected scaffold remains intact. Additionally, to clarify the mechanism of resistance change, cross-sectional SEM observations were performed near the boundary between the plasma-irradiated region and the untreated region (Figure S2). The plasma-irradiated region and the untreated region both showed almost no change in film thickness. This result suggests that O_2_ plasma irradiation modifies the local porous structure without reducing the macroscopic LIG film thickness; therefore, a decrease in electrical conductivity is considered to be the mechanism for resistance change.

These observations clarify the nature of “sub-visual defects” induced by the O_2_ plasma treatment. Specifically, the LIG undergoes pronounced electronic degradation, as evidenced by the resistance increase to *R*/*R*_0_ = 6.71 at 100 W, while the macroscopic structural scaffold remains largely intact. This discrepancy highlights the fundamental limitation of conventional optical or topographical inspections for evaluating LIG-based circuitry. Consequently, the results underscore the significance of our ML-ERT framework, which enables the spatial visualization of such invisible electrical failures through boundary potential sensing.

### 3.3 ML-ERT Reconstruction of Localized Defects

We subsequently evaluated the capability of our ML-ERT framework to spatially resolve the subvisual defects. Figure 4a displays a photograph of a representative circular LIG sample after localized 100 W plasma irradiation. As anticipated from the Raman and SEM analyses, the treated region remains indistinguishable from the pristine LIG under visible light, reinforcing the “invisible” nature of these localized electrical failures.

**Figure 4:**
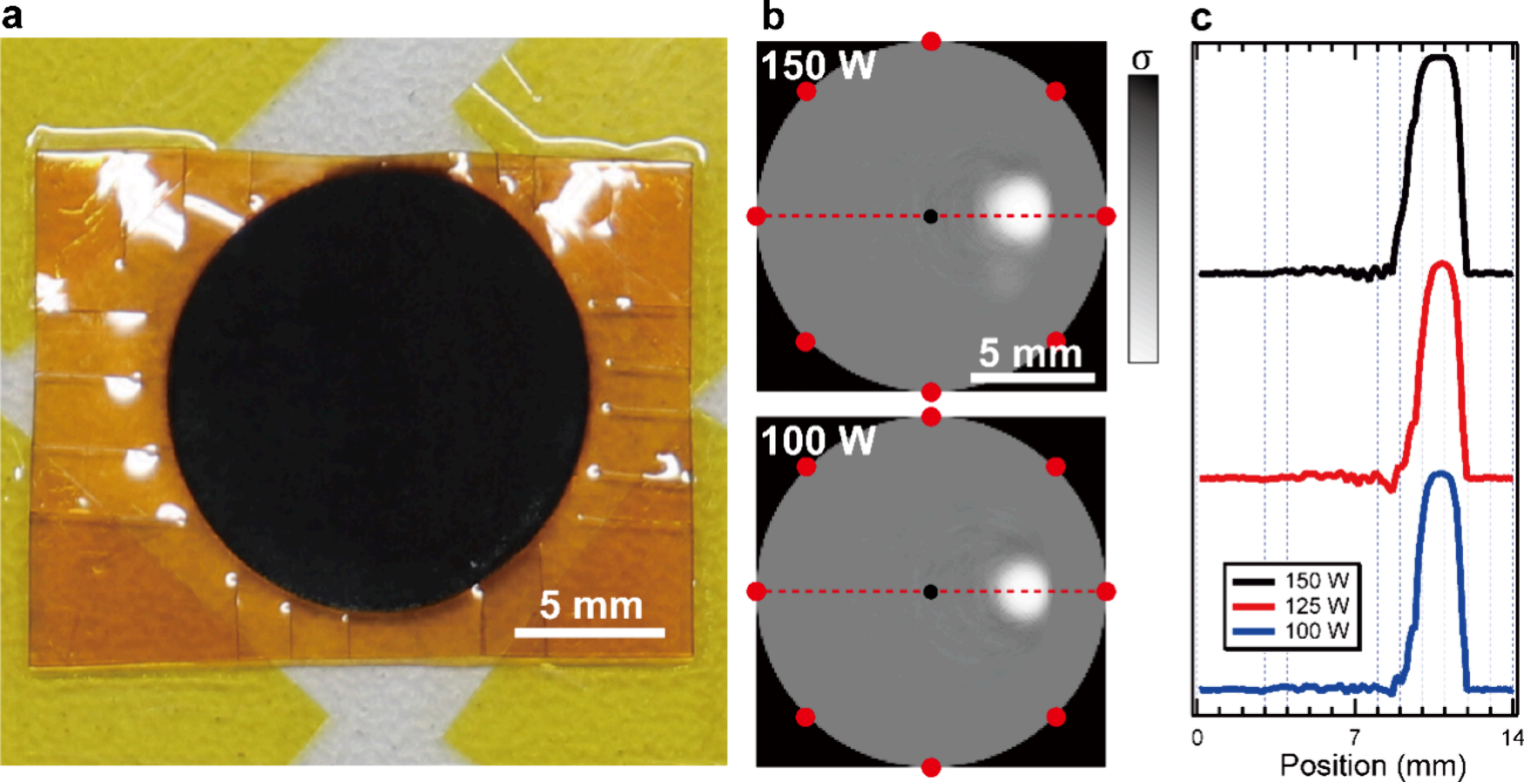
(a) Photograph of a circular LIG sample after localized O_2_ plasma irradiation (100 W). (b) Reconstructed conductivity distributions for LIG samples irradiated at 100 and 150 W, showing the localized defect region in each case. (c) Line profiles of the reconstructed conductivity along the dashed line indicated in (b).

Despite this lack of optical contrast, the ML-ERT successfully detected and localized the defect regions using only boundary voltage data. Figure 4b presents the reconstructed two-dimensional conductivity maps for samples treated at 100 and 150 W. In both cases, a localized conductivity dip is clearly visualized at the off-center position, precisely corresponding to the intended irradiation site. Reliable imaging was consistently achieved for plasma powers ≥ 100 W, which corresponds to a resistance-change ratio of *R*/*R*_0_ ≥ 6.71. This result defines the current sensitivity threshold of our model, demonstrating that even a conductivity contrast of approximately 1 order of magnitude is sufficient for robust tomographic localization.

To quantitatively assess the reconstruction accuracy, one-dimensional conductivity profiles were extracted across the defect center (Figure 4c). Based on the FWHM of the conductivity dip, the reconstructed defect diameters were estimated at 1.94 mm (100 W) and 2.36 mm (150 W). These values are moderately larger than the geometric aperture of the shadow mask (1.5 mm). This expansion of the defect footprint can be attributed to two main factors. First, from a physical standpoint, O_2_ plasma species can diffuse into the gap between the mask and the porous LIG surface, potentially causing a lateral “undercutting” effect that modifies the LIG slightly beyond the mask’s edge. Second, from a computational perspective, the ML-ERT reconstruction, which is similar to traditional ERT algorithms, tends to produce a spatially smoothed representation of sharp conductivity gradients, which may result in a slight overestimation of the defect size.^31^ Nevertheless, the consistent localization of these invisible regions demonstrates that the ML-ERT framework is a highly sensitive and effective tool for mapping functional degradation in LIG-based devices that would otherwise remain undetected by conventional inspection.

While the present study focuses on localized circular defects as a proof-of-concept, the ML-ERT framework is inherently scalable to more complex scenarios. To further examine the extensibility of the proposed framework beyond circular defects, an additional synthetic data set containing triangular defects was generated and used to retrain the model together with the original circular-defect data set. The retrained model successfully reconstructed triangular defect patterns from simulated boundary-voltage data, demonstrating that the present data-driven framework can be extended to noncircular defect geometries. These results are shown in Figure S3. Furthermore, increasing the electrode density would allow for higher spatial resolution, enabling the characterization of intricate defect patterns in large-scale LIG circuits.^47,48^ These results lay the groundwork for a versatile, real-time diagnostic platform for flexible carbon electronics.

### 3.4 3D FEM Analysis of Effective Thickness Reduction

To further elucidate the physical mechanism behind the ERT detection of subvisual defects, we conducted a three-dimensional FEM study. In this model, plasma-induced modifications are emulated as a localized reduction in the effective current-carrying thickness (Δ*t*) of the LIG film, rather than a mere change in surface topography. The simulation geometry, featuring eight electrodes on a 10 mm circular domain (Figure 5a,b), directly mirrors our experimental setup, with a baseline LIG thickness of 50 μm.

**Figure 5:**
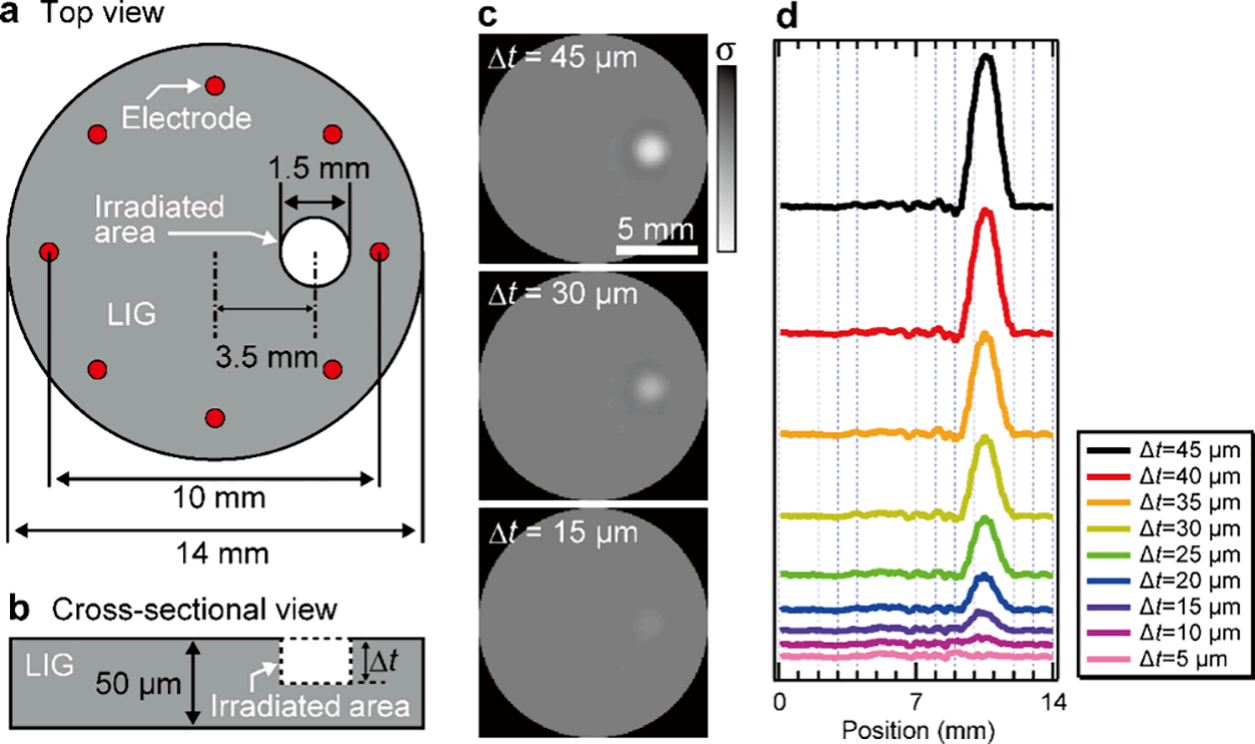
(a) Top-view schematic of the simulation geometry, reproducing the experimental sample and electrode arrangement (the irradiated region is positioned according to the mask design). (b) Cross-sectional schematic of the thickness-reduction model: the baseline LIG film thickness is 50 μm, and the plasma-affected region is modeled as a local reduction in effective thickness by Δ*t*. (c) Reconstructed conductivity maps for representative Δ*t* values, showing increasing defect contrast with larger thickness loss. (d) Line profiles across the defect for Δ*t* = 5–45 μm, illustrating the monotonic increase in reconstruction contrast with increasing Δ*t*.

Figure 5c illustrates the evolution of the reconstructed conductivity maps as a function of Δ*t*. As the local conductive cross-section is restricted, the reconstructed contrast at the defect site becomes increasingly pronounced. Our analysis reveals that a clear defect visualization is achieved when Δ*t* ≥ 15 μm (Figure 5d), corresponding to an approximately 30% reduction in effective thickness in the present model. It should be noted that the ∼30% reduction in effective thickness is not intended to represent a universal theoretical threshold. Rather, it is a value obtained within the present 3D FEM model, at which the reconstructed ERT contrast became sufficiently clear. Therefore, the thickness reduction parameter should be interpreted as a simplified proxy for the loss of internal conductive pathways rather than as direct evidence of a percolation-theory-based critical transition.

Crucially, the parameter Δ*t* in this model represents the loss of internal conductive pathways within the 3D porous network, rather than a uniform physical thinning of the film. This interpretation explains why the ERT can resolve significant conductivity anomalies even when the surface appears morphologically intact under SEM or optical inspection. By bridging the gap between microscopic pathway degradation and macroscopic potential distribution, this FEM study provides a robust physical foundation for the high sensitivity of our ML-ERT framework in detecting subsurface electronic failures.

### 3.5 Origin of Plasma-Induced Conductivity Contrast

The aforementioned results demonstrate that ML-ERT can accurately localize plasma-induced defects in porous LIG even when the modification is visually imperceptible. This implies that the detectability of a defect is governed primarily by electrically meaningful perturbations within the internal conductive network, rather than by macroscopic morphological changes. Figure 6 provides a schematic conceptualization of this phenomenon.

**Figure 6:**
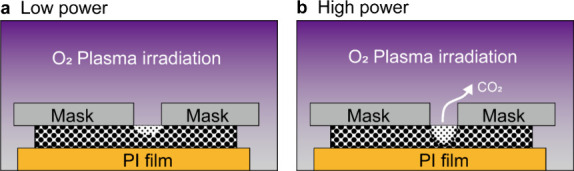
(a) Low-power plasma irradiation: modification is limited to the near-surface region and around the mask edges, and the conductive network remains largely intact. (b) High-power irradiation: enhanced etching of the porous network (including partial under-mask erosion) reduces the effective current-carrying pathways/volume, generating a much higher conductivity contrast that is detectable by ERT.

Under low-power plasma irradiation (Figure 6a), reactive species primarily introduce structural disorder near the surface and along the mask edges, while the underlying conductive network remains largely intact. Consequently, the global conductivity suffers only minor suppression, yielding limited contrast in the reconstructed ERT images. In contrast, high-power irradiation (Figure 6b) facilitates more aggressive ion bombardment and chemical etching, which not only erodes the porous carbon framework but also penetrates deeper into the scaffold. This process effectively disrupts the internal conductive pathways, leading to a substantial loss of conductive volume and generating the pronounced conductivity contrast required for robust ERT detection.

The 3D FEM analysis in Section 3.4 corroborates this interpretation by quantifying how a reduction in the effective current-carrying cross-section dramatically enhances reconstruction contrast. In this context, the thickness reduction parameter Δ*t* should be viewed as a proxy for the cumulative degradation of internal pathways rather than a uniform geometric thinning. Collectively, these findings establish that ML-ERT is an effective tool for the nondestructive localization of electrically degraded regions in LIG-based electronics. However, it should also be noted that the present results do not provide direct nanoscale proof of the percolation pathway hypothesis. Rather, this interpretation is based on the combined observations that ERT visualization required a sufficiently large resistance change (*R*/*R*_0_ ≥ 6.71), Raman spectroscopy indicated structural disorder, SEM did not show a correspondingly large macroscopic geometrical loss, and the 3D FEM model reproduced the observed ERT contrast when the effective conductive cross-section was reduced. It should be noted, however, that ML-ERT provides the spatial distribution of conductivity degradation, but does not by itself distinguish the specific origin of that degradation, such as structural discontinuity, oxidation, or lattice defects. Therefore, identification of the underlying mechanism requires complementary characterization methods, such as Raman spectroscopy, SEM, and chemical analyses including EDS or XPS.

## 4 Conclusions

In summary, we have successfully demonstrated an ML-ERT framework for the nondestructive localization of subvisual defects in porous laser-induced graphene. While oxygen plasma irradiation significantly degrades the electrical conductivity of LIG, the degraded regions were difficult to be distinguished by naked-eye observation and low-magnification top-view SEM image. In the present system, ML-ERT reliably localized such regions when the resistance contrast reached *R*/*R*_0_ ≥ 6.71. By integrating eight boundary electrodes with a 1D-CNN-based inverse solver, we reconstructed two-dimensional conductivity maps that localized these electrically degraded regions.

The 3D FEM analysis and structural characterizations further elucidated that the observed ERT contrast originates from the effective loss of internal conductive pathways, which represents a loss of conductive volume rather than a geometrically evident thinning of the material. These findings highlight the fundamental advantage of ML-ERT in detecting functional degradation that escape surface-based diagnostic tools. Although this work focused on circular defects as a proof-of-concept, the data-driven nature of our ML approach allows for future expansion toward detecting irregular damage patterns and multiple defect sites in large-area electronics. Overall, our ML-ERT framework provides a rapid, high-sensitivity, and practical solution for the process monitoring and quality control of advanced carbon-based conductive films, thereby bridging the gap between subsurface electronic failure and noninvasive spatial diagnostics.

## Supplementary Material



## Data Availability

The data sets used and/or analyzed during the current study are available from the corresponding author on reasonable request. The Python code used for the 1D-CNN training and conductivity-map reconstruction is publicly available on GitHub in the 2026/KM_ACSOmega directory of the following repository: https://github.com/TUS-ikunolab/public_data.git.

## Notes

Training behavior of the 1D-CNN model, cross-sectional SEM image of plasma-treated LIG, and reconstruction result for a triangular defect using a retrained 1D-CNN model (PDF)
