# Supplementary material for: Nondestructive Localization of Subvisual Defects in Laser-Induced Graphene via Machine-Learning-Assisted Electrical Resistance Tomography
Source: ACS Omega. 2026 Jun 4;11(23):34287–94. doi: 10.1021/acsomega.6c01789 (PMC13281016; doi:10.1021/acsomega.6c01789)
Supplement: Supplementary file 1 [file ao6c01789_si_001.pdf]

Supplementary information

Non-destructive Localization of Sub-visual Defects in Laser-Induced Graphene via Machine-Learning-Assisted Electrical Resistance Tomography

Keiya Minakawa[1], Kotaro Takanashi[1], Yuki Kimura[1], Annop Klamchuen[2], Winadda Wongwiriyapan[3], and Takashi Ikuno[1,4*]

1. Department of Applied Electronics, Graduate School of Advanced Engineering, Tokyo University of Science, Katsushika, Tokyo 125-8585, Japan

2. National Nanotechnology Center, NSTDA, 111 Thailand Science Park, Klong Luang, Pathum Thani 12120, Thailand

3. Department of Nanoscience and Nanotechnology, School of Integrated Innovative Technology, King Mongkut's Institute of Technology Ladkrabang, College of Materials Innovation and Technology, Ladkrabang, Bangkok 10520, Thailand

4. Research Institute for Science and Technology, Tokyo University of Science, Noda, Chiba 278-8510, Japan

*tikuno@rs.tus.ac.jp

To monitor the training process of the 1D-CNN, the MSE and MAE were tracked over 100 epochs, as shown in Figure S1a and S1b, respectively. Both metrics decreased steadily during training, indicating stable optimization and convergence on the full synthetic dataset. It should be noted that no separate validation or test dataset was used in this study; therefore, these results demonstrate training behavior rather than rigorous predictive performance on unseen data.

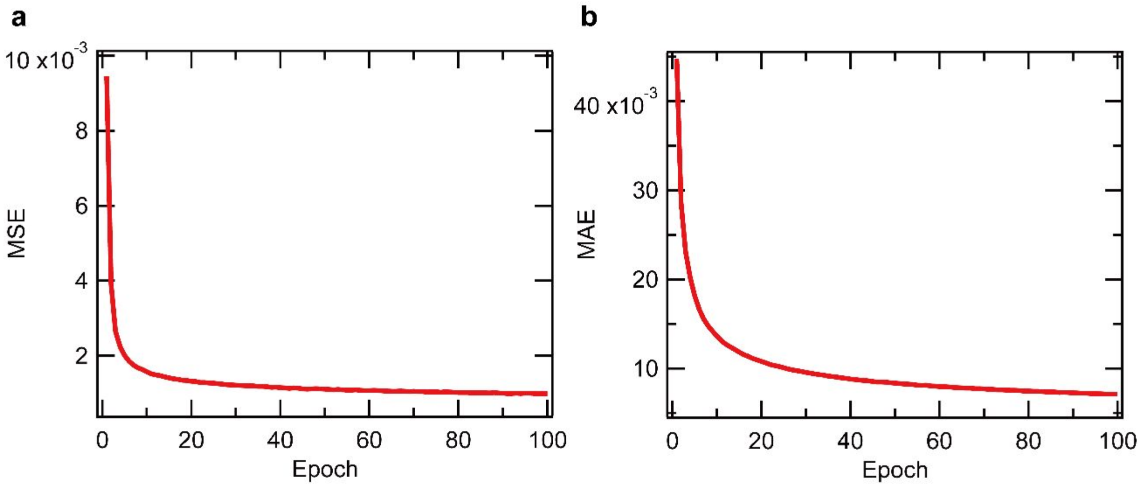

Figure S1. Training behavior of the 1D-CNN model using the full synthetic dataset. (a) MSE and (b) MAE plotted as functions of training epoch. Both metrics decreased monotonically during training, indicating convergence on the training dataset.

Figure S2 shows a cross-sectional SEM image near the boundary between the plasma irradiated region and pristine region of the LIG after O_2 plasma treatment. The cross-sectional view reveals a difference in the surface/near-surface morphology across the boundary. Importantly, no obvious macroscopic reduction in the overall LIG thickness is observed. These results suggest that the plasma treatment modifies the local porous microstructure without causing a large geometrical thinning of the entire LIG layer.

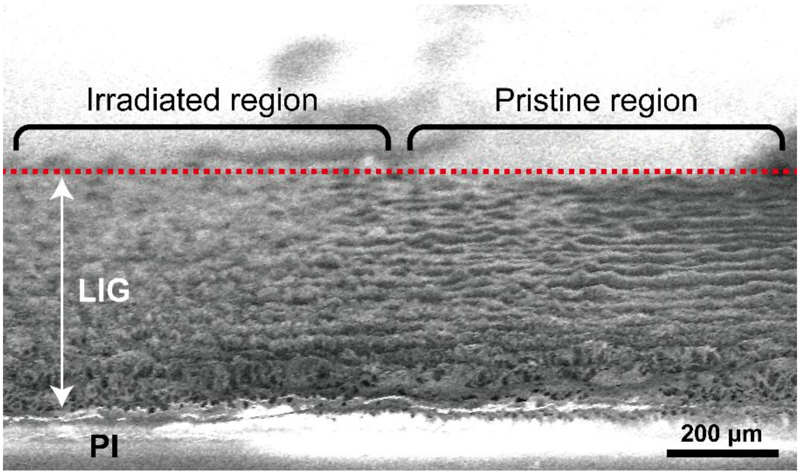

Figure S2. Cross-sectional SEM image of LIG near the boundary between the plasma irradiated region (left) and the pristine region (right) after O_2 plasma treatment. Although no obvious macroscopic reduction in the overall LIG thickness is observed, a distinguishable difference in the surface/near-surface morphology can be seen between the two regions.

To examine whether the proposed ML-ERT framework can be extended beyond circular defects, an additional synthetic dataset containing triangular defects was generated and combined with the original circular-defect dataset. A new 1D-CNN model was then trained on the combined dataset under the same training conditions as the original model (100 epochs, batch size = 60). **Figure S3a** shows the simulated conductivity distribution containing a triangular defect, and **Fig. S3b** shows the corresponding reconstructed conductivity map obtained from simulated boundary voltage data. The successful localization of the triangular defect suggests that the proposed data-driven framework is extensible to non-circular defect geometries.

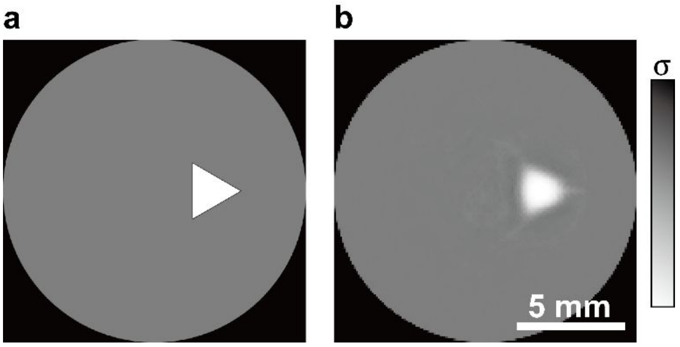

Figure S3. Reconstruction of a triangular defect using the retrained 1D-CNN model. (a) Simulated conductivity distribution with a triangular defect. (b) Conductivity map reconstructed from simulated boundary voltage data.
